# GPR68 Inhibition Induces Ferroptosis in Atypical Teratoid Rhabdoid Tumors

**DOI:** 10.1002/cnr2.70719

**Published:** 2026-10-16

**Authors:** Leif R. Neitzel, Charles C. Hong

**Affiliations:** ^1^ Department of Medicine Michigan State University College of Human Medicine East Lansing Michigan USA; ^2^ Henry Ford Health + Michigan State Health Sciences Detroit Michigan USA

**Keywords:** ATRT, atypical teratoid rhabdoid tumor, GPR68, OGM, OGR1, ogremorphin

## Abstract

**Background:**

Atypical teratoid/rhabdoid tumors (ATRTs) are aggressive pediatric CNS malignancies with < 30% 5‐year survival despite multimodal therapy. No prior studies have explored ferroptosis induction in ATRT.

**Aims:**

To determine whether GPR68 inhibition by ogremorphin (OGM) induces *ATF4*‐dependent ferroptosis in ATRT cell lines and whether SLC7A11 blockade (erastin, ERA) confers similar sensitivity.

**Methods:**

CHLA‐02‐ATRT and CHLA‐06‐ATRT cell lines were treated with OGM or ERA. Viability (CellTiter‐Glo), GSH levels (GSH‐Glo), lipid peroxidation (BODIPY 581/591 C11), and gene expression (qRT‐PCR: *ATF4*, *HMOX1*, *SLC7A11*, *TFRC*) were assessed. Genetic validation used GPR68/ATF4 shRNA, overexpression, and an *ATF4* luciferase reporter. Statistics: Student's *t*‐test with Bonferroni correction (R 4.4.0).

**Results:**

Both cell lines were sensitive to OGM‐ and ERA‐induced ferroptosis, with CHLA‐02‐ATRT significantly more responsive to OGM than ERA. OGM/ERA depleted GSH, increased lipid peroxidation, and upregulated *ATF4*, *HMOX1*, and *SLC7A11* mRNA expression. OGM induced *TFRC* in both cell lines; high‐dose ERA upregulated *TFRC* in CHLA‐02‐ATRT only. GPR68 knockdown reduced viability, while overexpression or Ogerin increased proliferation. OGM activated *ATF4* transcription, and *ATF4* overexpression decreased cell survival. *ATF4* knockdown rescued OGM‐treated cells.

**Conclusion:**

GPR68 inhibition triggers *ATF4*‐dependent ferroptosis in ATRT. SLC7A11 blockade by ERA paralleled the effects of ferroptosis by OGM. These data establish a novel therapeutic strategy with strong translational potential.

**Abbreviations**
ATRT: atypical teratoid/rhabdoid tumor
CNS: central nervous system
ERA: erastin
GSH: glutathione
OGM: ogremorphin
OGR‐1: ovarian cancer G protein‐coupled receptor 1
qRT‐PCR: quantitative reverse transcription PCR
TME: tumor microenvironment

## 1 Introduction

Atypical teratoid/rhabdoid tumors (ATRTs) are highly malignant central nervous system (CNS) neoplasms predominantly affecting young children, with over 90% of cases diagnosed before age 3 years [1, 2]. These tumors exhibit multi‐lineage differentiation and a primitive pathological phenotype [2]. Although ATRTs comprise only 1%–5% of all pediatric CNS tumors, they account for approximately 20% of embryonal CNS tumors and up to 50% of CNS malignancies in the first year of life [3, 4, 5, 6, 7, 8, 9]. ATRTs primarily arise infratentorially, with over 60% in the posterior fossa, though location varies by age: Posterior fossa tumors predominate in infants (< 1 year), supratentorial tumors in toddlers, and spinal tumors in children ≥ 3 years [10, 11, 12, 13, 14]. The mean age at diagnosis is 16–28 months, predominantly in males (male‐to‐female ratio: 1.1–2:1) [1, 4, 8, 11, 15, 16, 17, 18]. Rare cases occur in adolescents and adults [5, 19]. Treatment is typically bi‐ or trimodal, tailored to patient age, molecular subtype and metastatic status, and consists of maximal safe surgical resection followed by intensive chemotherapy and radiotherapy [20, 21]. Despite aggressive multimodal approaches, outcomes remain dismal: Median overall survival is 16.9 months (range: 6–18 months across cohorts), with 5‐year overall survival < 30% [9, 22, 23, 24]. These poor prognoses underscore the urgent need for novel, targeted therapies to disrupt key survival pathways in ATRT.

Ogremorphin (OGM), a specific selective inhibitor of the proton‐sensing G protein‐coupled receptor 68 (GPR68; also known as ovarian cancer G protein‐coupled receptor 1 or OGR‐1), induces ferroptosis through disruption of the GPR68/ATF4 pro‐survival axis in glioblastoma, diffuse intrinsic pontine glioma (DIPG), lung, and pancreatic cancers [25, 26, 27]. OGM demonstrates efficacy both in vitro and in vivo [25, 26, 27, 28, 29]. GPR68 is activated by acidification of the tumor microenvironment (TME; pH 6.4–7.4), a hallmark of aggressive malignancies driven by the Warburg effect, which promotes radioresistance, immune evasion, and metastatic potential [30, 31, 32, 33, 34, 35, 36, 37, 38, 39, 40]. OGM not only triggers ferroptosis but also sensitizes glioblastoma, lung, and pancreatic cells to radiotherapy [25, 26].

Mirroring glioblastoma, ATRTs are treated with maximal safe resection followed by intensive chemotherapy and radiotherapy, yet outcomes remain dismal. Despite shared reliance on an acidic TME and radioresistance, no studies to date have investigated ferroptosis induction in ATRTs. Herein, we demonstrate that GPR68 inhibition by OGM induces ATF4‐dependent ferroptotic cell death in ATRT cell lines. Furthermore, we demonstrate that ATRTs are sensitive to SLC7A11 blockade by erastin (ERA), a second GPR68‐independent form of ferroptosis induction. These data present a novel, translatable therapeutic axis for this lethal pediatric brain tumor.

## 2 Results

### 2.1 ATRT Cells Exhibit Differential Sensitivity to OGM and ERA

We first evaluated ferroptosis sensitivity in two well‐characterized atypical teratoid rhabdoid tumors cell lines: CHLA‐02‐ATRT and CHLA‐06‐ATRT. CHLA‐02‐ATRT was derived from a resected brain tumor of a 1‐year‐old male patient, while CHLA‐06‐ATRT originated from the cerebellum of a 4‐month‐old female patient. Ferroptosis is an iron‐dependent form of programmed cell death driven by lipid peroxidation. OGM, a specific GPR68 inhibitor, induces ATF4 expression and promotes ferroptotic cell death. ERA is a well‐validated ferroptosis inducer and distinct mechanistic comparator that served as a positive control. ERA inhibits SLC7A11, a subunit of the cystine/glutamate antiporter system Xc^−^ (with SLC3A2), thereby blocking cysteine uptake and depleting glutathione (GSH). Reduced GSH impairs GPX4‐mediated detoxification of lipid hydroperoxides, culminating in ferroptosis.

Both CHLA‐02‐ATRT and CHLA‐06‐ATRT cells were sensitive to OGM‐ and ERA‐induced ferroptosis (Figure 1A,B). Notably, CHLA‐02‐ATRT exhibited greater sensitivity to OGM than to ERA (Figure 1A), conversely, CHLA‐06‐ATRT showed comparable sensitivity to both compounds (Figure 1B). In contrast, the GPR68 positive allosteric modulator, Ogerin, enhanced proliferation in both cell lines (Figure 1C). GPR68 mRNA expression was significantly lower in CHLA‐02‐ATRT and CHLA‐06‐ATRT compared to the glioblastoma cell line U138MG, in which OGM‐induced ferroptosis is well‐established (Figure 1D). These data suggest that basal GPR68 activity supports ATRT cell growth and that inhibition of either GPR68 or SLC7A11 promotes ferroptosis in ATRT cells.

**FIGURE 1:**
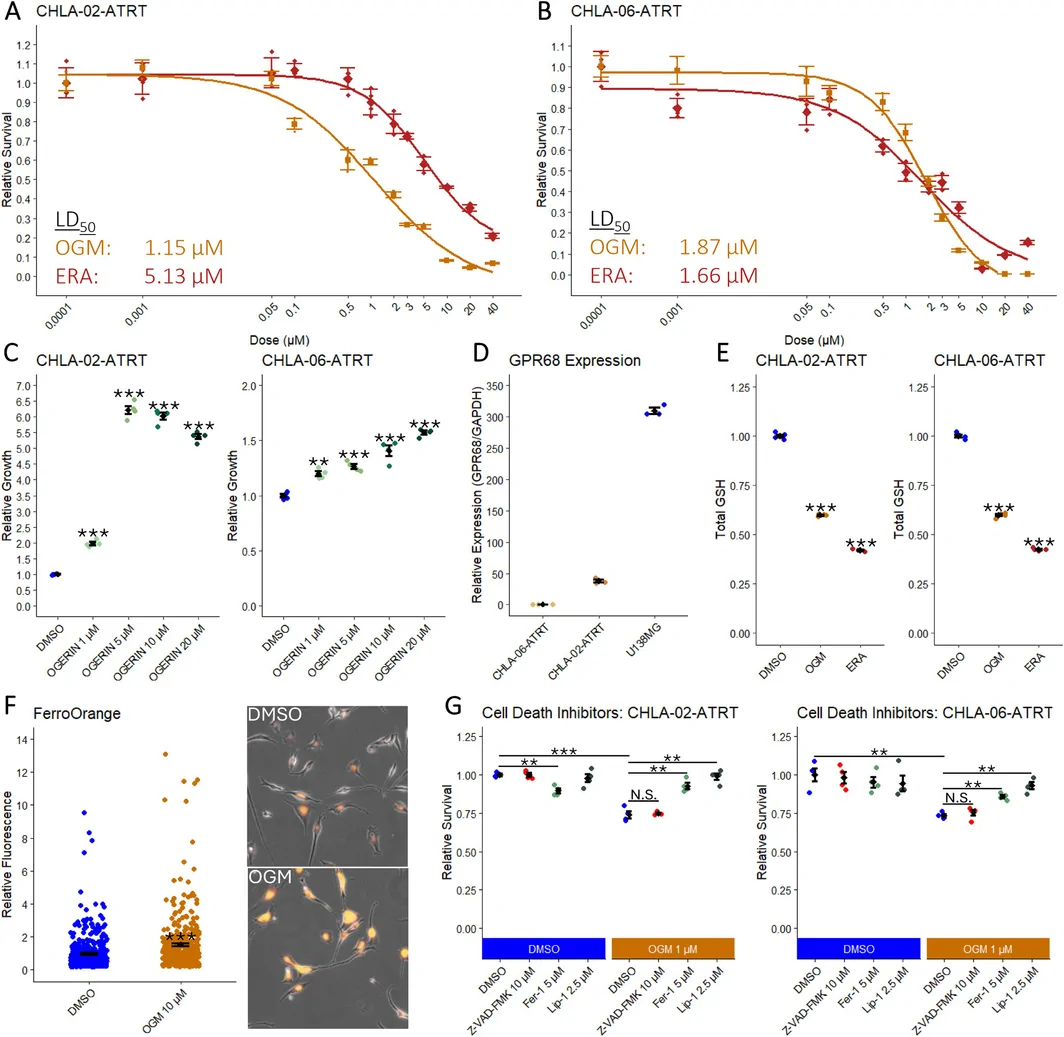
ATRT cell lines are sensitive to ferroptosis inducers. Survival curves for (A) CHLA‐02‐ATRT and (B) CHLA‐06‐ATRT lines treated with OGM compared to the positive control ERA. (C) Treatment with the selective positive allosteric modulator of GPR68, Ogerin, increased the growth of both ATRT cell lines. (D) Expression of GPR68 in both ATRT cell lines compared to the highly expressing glioblastoma positive control cell line U138MG. (E) Treatment with either OGM or ERA decreased total glutathione levels in both ATRT cell lines. (F) FerroOrange staining of adherent CHLA‐06‐ATRT cells shows increased ferrous iron (Fe^2+^) in OGM treated samples. (Right) representative images. (G) Cotreatment with the ferroptosis inhibitors ferrostatin‐1 (Fer‐1) and liproxstatin‐1 (Lip‐1) rescues OGM mediated ferroptosis. Conversely, Z‐VAD‐FMK, a pan‐caspase inhibitor of apoptosis, had no effect on OGM‐mediated cell death. (A), (B), and (C) *n* = 6 biological repeats with *n* = 4 technical repeats. (D) *n* = 3 biological repeats with *n* = 3 technical repeats. (E) *n* = 4 biological repeats with *n* = 4 technical repeats and 2 μM OGM or 5 μM ERA. (F) *n* ≥ 368 cells aggregated from *n* = 3 biological repeats. (G) *n* = 3 biological repeats with *n* = 4 technical repeats. (A), (B), (C), and (G) mean ± SD with significance determined by two‐tailed, equal variance, *t*‐tests. (F) mean ± SEM with significance determined by two‐tailed, equal variance, *t*‐tests with ***p < 0.001. (C) Comparisons are between DMSO and treatments **p < 0.0025 and ***p < 0.00025. (E) Comparisons are between DMSO and treatments ***p < 0.0005. (G) Seven comparisons were made between DMSO and OGM controls, and DMSO or OGM co‐treatments with Z‐VAD‐FMK, Fer‐1, or Lip‐1 **p < 0.00143 and ***p < 0.000143.

### 2.2 OGM and ERA Induce Ferroptosis in ATRT Cells

OGM‐mediated ferroptosis is well‐established in glioblastoma, lung carcinoma, and pancreatic ductal adenocarcinoma cells. To confirm that OGM and ERA induce ferroptosis in ATRT, we assessed key biochemical and transcriptional hallmarks. Depletion of intracellular GSH, a critical substrate for GPX4‐mediated detoxification of lipid hydroperoxides, was observed in both CHLA‐02‐ATRT and CHLA‐06‐ATRT cells following OGM or ERA treatment (Figure 1E). FerroOrange, a cell‐permeable fluorescent probe, selectively and irreversibly binds intracellular ferrous iron (Fe^2+^) in live cells. In adherent CHLA‐06‐ATRT cells, OGM upregulated intracellular ferrous iron levels, consistent with ferroptosis (Figure 1F). Rescue with the ferroptosis inhibitors ferrostatin‐1 (Fer‐1) and liproxstatin‐1 (Lip‐1) is considered the gold‐standard validation of ferroptosis. Both Fer‐1 and Lip‐1 rescued OGM‐mediated cell death in CHLA‐02‐ATRT and CHLA‐06‐ATRT cells (Figure 1G). In contrast, the pan‐caspase inhibitor Z‐VAD‐FMK had no effect on OGM‐induced loss of viability (Figure 1G).

We next used qRT‐PCR to assess four markers of OGM‐induced ferroptosis: ATF4, HMOX1, SLC7A11, and TFRC. Although no universal ferroptosis reporters exist and ERA‐induced transcriptional changes are context‐dependent, both OGM and ERA upregulated *ATF4*, *HMOX1*, and *SLC7A11* in the two ATRT lines (Figure 2A–C). OGM induced *TFRC* expression in both cell lines, whereas high‐dose ERA increased *TFRC* in CHLA‐02‐ATRT but reduced it in CHLA‐06‐ATRT (Figure 2D). Comparisons between the housekeeping genes GAPDH and ACTB showed that OGM and ERA do not alter GAPDH expression in either CHLA‐02‐ATRT or CHLA‐06‐ATRT cell lines (Figure 2E). Together, these data confirm that OGM‐ and ERA‐mediated ferroptosis in ATRT cells.

**FIGURE 2:**
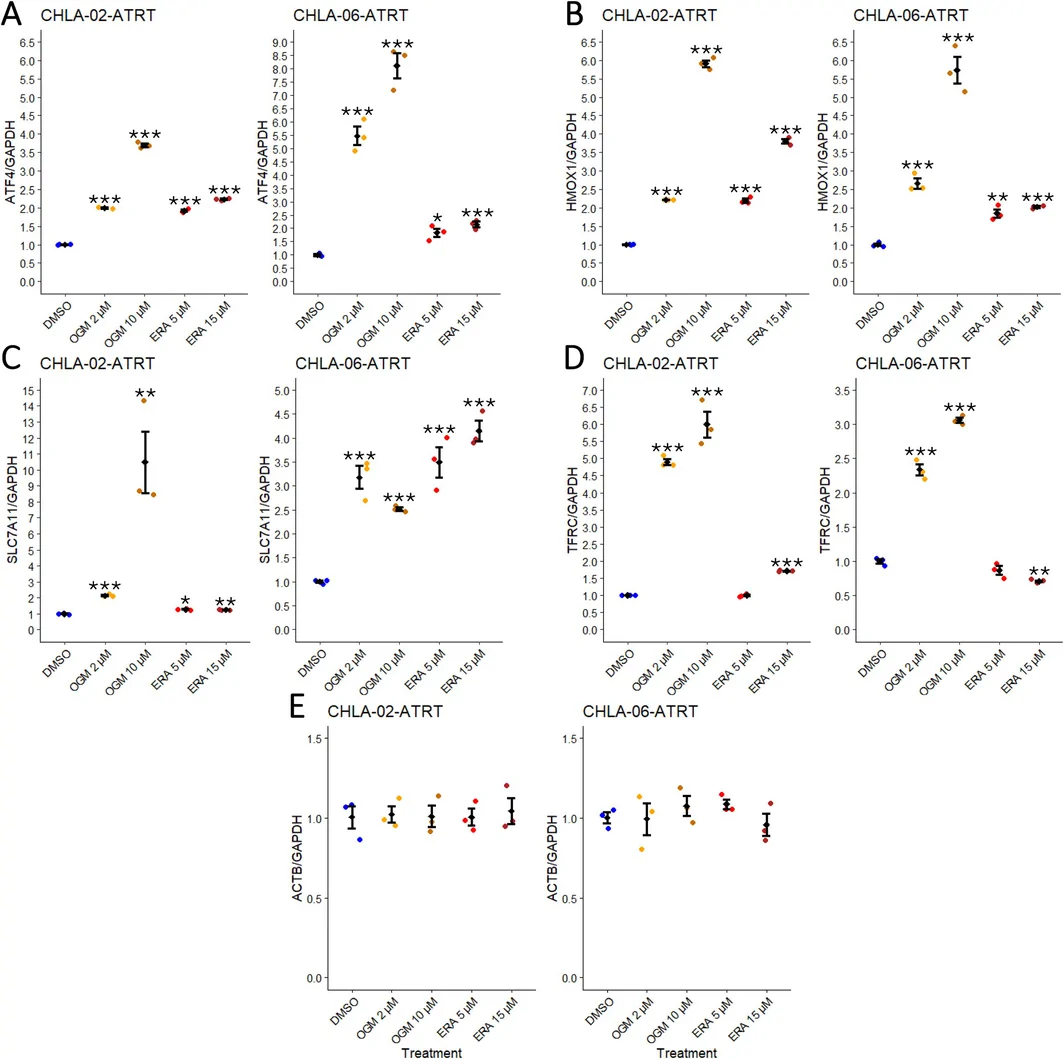
OGM and ERA induce ferroptosis reporter genes in ATRT cell lines. Relative expression of known OGM‐mediated ferroptosis reporter genes (A) ATF4, (B) HMOX1, (C) SLC7A11, and (D) TFRC in CHLA‐02‐ATRT and CHLA‐06‐ATRT lines treated with OGM or ERA. (E) Relative expression of a second housekeeping gene, ACTB, confirming that GAPDH expression is unaffected by OGM or ERA. (A–E) *n* = 3 biological repeats with *n* = 3 technical repeats. Graphs represent mean ± SD with significance determined by two‐tailed, equal variance, *t*‐tests. Comparisons are between DMSO and treatments *p < 0.0125, **p < 0.0025, and ***p < 0.00025.

CHLA‐02‐ATRT cells grow in suspension, whereas CHLA‐06‐ATRT cells exhibit a mixed adherent and suspension phenotype, with fetal bovine serum supplementation promoting adherence in the latter [41]. To further validate ferroptosis induction by OGM and ERA, adherent CHLA‐06‐ATRT cells were stained with BODIPY 581/591 C11, a ratiometric fluorescent sensor of lipid peroxidation. Upon oxidation, BODIPY 581/591 C11 shifts from red to green fluorescence. Both OGM and ERA exhibited increased dye oxidation, indicating elevated lipid hydroperoxide levels (Figure 3A,B). This OGM‐ and ERA‐induced lipid peroxidation supports ferroptosis as the mechanism of cell death in ATRT cells.

**FIGURE 3:**
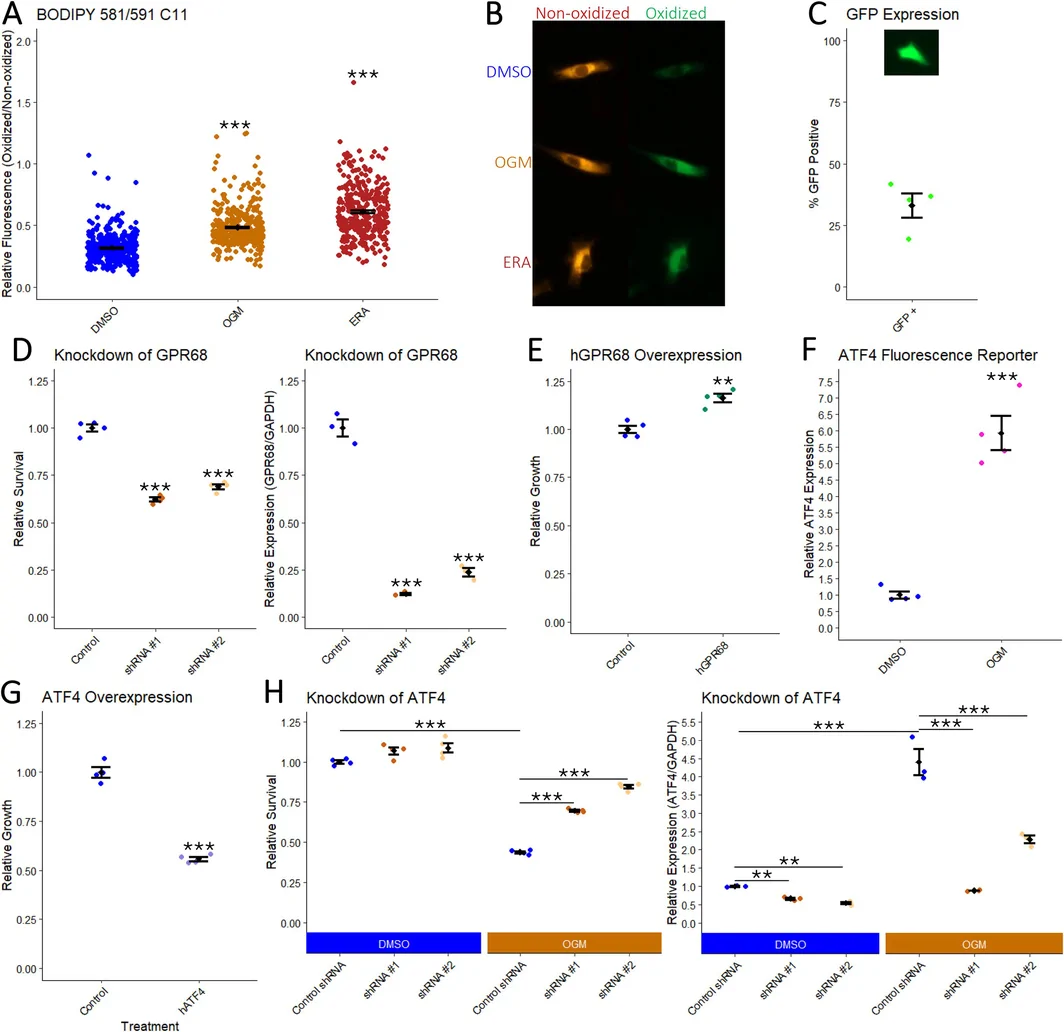
Genetic validation of ferroptosis in CHLA‐06‐ATRT. (A) Treatment with OGM or ERA increases Bodipy 581/591 C11 oxidation, indicating increased lipid peroxidation. (B) Representative images from (A). (C) Expression of GFP demonstrates the transfectability of CHLA‐06‐ATRT cells. (D) Transfection with siRNA against GPR68 decreases cell viability (left) and the relative expression of GPR68 (right). (E) Overexpression of human GPR68 increases cell growth. (F) An ATF4 reporter plasmid indicates that treatment with OGM increases ATF4 expression. (G) Overexpression of human ATF4 is sufficient to decrease cell survival. (H) Transfection with siRNA against ATF4 rescues cell viability (left) and decreases the relative expression of ATF4 (right). (A) *n* ≥ 4 biological repeats with *n* ≥ 355 aggregated cells and 10 μM OGM or 15 μM ERA. (C) *n* = 4 biological repeats. (D) and (H) For cell survival, *n* = 8 biological repeats with *n* = 4 technical repeats, and for qRT‐PCR *n* = 3 biological repeats with *n* = 3 technical repeats. (H) 2 μM OGM. (E) and (G) *n* = 8 biological repeats with *n* = 4 technical repeats. (F) *n* = 6 biological repeats with *n* = 4 technical repeats and 2 μM OGM. (A) Mean ± SEM with significance determined by two‐tailed, equal variance, *t*‐tests with ***p < 0.0005. (B, C) ×20 magnification. (D–H) Mean ± SD with significance determined by two‐tailed, equal variance, t‐tests. (D) Comparisons are between control and treatments ***p < 0.0005. (E–G) **p < 0.01 and ***p < 0.001. (H) Five comparisons are between control and treatments, and **p < 0.002 and ***p < 0.0002.

### 2.3 GPR68 Inhibition Induces ATF4‐Dependent Ferroptosis in ATRT Cells

To validate the effects of OGM, we wanted to recapitulate the effects of genetic inhibition of GPR68 as seen in glioblastoma, lung, and pancreatic cancer [25, 26]. First, we validated the transfectability of adherent CHLA‐06‐ATRT cells using a GFP expression vector (Figure 3C). Transfection of shRNA against *GPR68* reduced mRNA levels and cell viability in CHLA‐06‐ATRT cells (Figure 3D). Conversely, *GPR68* overexpression enhanced proliferation, mirroring the effects of the GPR68 positive allosteric modulator Ogerin (Figure 3E). These findings demonstrate that genetic manipulation of GPR68 recapitulates the effects of OGM and Ogerin on ATRT cell survival.

GPR68 suppresses ferroptosis by inhibiting *ATF4* expression in glioblastoma, lung, and pancreatic cancer cells. To test this in ATRT, we first used an *ATF4* reporter plasmid, which confirmed that OGM induces *ATF4* transcriptional activity, consistent with qRT‐PCR data (Figures 2A and 3F). Furthermore, *ATF4* overexpression was sufficient to induce cell death in CHLA‐06‐ATRT cells (Figure 3G). As *ATF4* inhibition rescues OGM‐induced ferroptosis in other cancers, we tested this using *ATF4*‐targeting shRNAs in OGM‐treated CHLA‐06‐ATRT cells. *ATF4* knockdown reduced *ATF4* mRNA expression and increased cell viability (Figure 3H). These data suggest that OGM‐induced ferroptosis in ATRT cells is *ATF4*‐dependent, consistent with findings in glioblastoma, lung, and pancreatic cancers.

## 3 Discussion

Our findings demonstrate that GPR68 inhibition induces ATF4‐dependent ferroptosis in atypical teratoid rhabdoid tumor cells, mirroring mechanisms observed in glioblastoma, lung, and pancreatic cancers. Both CHLA‐02‐ATRT and CHLA‐06‐ATRT cells exhibited sensitivity to OGM and ERA mediated ferroptosis. This was confirmed by multiple ferroptosis hallmarks: GSH depletion, lipid peroxidation, and upregulation of ferroptosis‐associated genes (*ATF4*, *HMOX1*, *SLC7A11*, *TFRC*) [25, 26]. While OGM inhibits the proton‐sensing GPCR GPR68, ERA blocks cystine import via system X_c_^−^. Both ultimately deplete GSH and induce ferroptosis, but they do so through distinct upstream pathways and molecular targets. We further validated that OGM induces ferroptosis by demonstrating ferrous iron accumulation and by showing that the ferroptosis inhibitors Fer‐1 and Lip‐1 rescue OGM‐mediated cell death, whereas the pan‐caspase inhibitor Z‐VAD‐FMK has no effect. Notably, CHLA‐02‐ATRT showed greater sensitivity to OGM than ERA. However, CHLA‐06‐ATRT displayed comparable sensitivity to both compounds. This cell line‐specific difference was further reflected in the expression of *TFRC* mRNA: High‐dose ERA upregulated TFRC in CHLA‐02‐ATRT but slightly downregulated it in CHLA‐06‐ATRT. These variations in the ATRT cell lines may reflect their distinct molecular profiles, including lower basal GPR68 expression in CHLA‐06‐ATRT compared to CHLA‐02‐ATRT, potentially influencing therapeutic responsiveness.

The GPR68/ATF4‐dependent ferroptosis axis recapitulates that seen in glioblastoma, lung, and pancreatic cancers. OGM robustly induced ATF4 transcription and activity, validated by a reporter assay and qRT‐PCR. Conversely, ATF4 knockdown rescued viability in OGM‐treated CHLA‐06‐ATRT cells. These results align with prior studies suggesting that GPR68 suppresses ferroptosis by inhibiting ATF4 [25, 26].

Genetic manipulation of GPR68 recapitulates the effects of OGM and Ogerin, further confirming ferroptosis as the primary cell death mechanism. shRNA‐mediated knockdown of GPR68 reduced the viability of CHLA‐06‐ATRT. Ogerin‐mediated agonism of GPR68 enhanced the proliferation of both CHLA‐02‐ATRT and CHLA‐06‐ATRT cell lines. This finding was further bolstered by the overexpression of GPR68, which increased CHLA‐06‐ATRT proliferation. These findings underscore GPR68 as a potential driver of ATRT growth and survival. Therefore, the induction of ferroptosis via GPR68 inhibition offers a novel strategy for reducing both ATRT cell viability and growth.

These findings present significant therapeutic implications for ATRT, a highly aggressive and devastating pediatric brain tumor with limited treatment options and dismal prognosis. Our data establish GPR68 inhibition as a promising ferroptosis‐based therapeutic strategy for ATRT, with ATF4 as a key mediator. Additional data on SLC7A11 blockade (ERA) broadens the scope of ferroptosis inducers as potential therapeutic treatments of ATRT. These data warrant further studies with priority on in vivo models and combinational therapies to enhance clinical translatability.

## 4 Methods

### 4.1 Cell Culture

ATRT cells were obtained from the American Type Culture Collection (ATCC) and maintained in DMEM:F12 with GlutaMAX (Gibco 10565018) supplemented with 10 mL B‐27 Supplement (Gibco, 17504‐044), 1 mL of 10 μg/mL EGF resuspended in PBS with 0.1% BSA (Gibco, PHG0311), 1 mL of 10 μg/mL FGF resuspended in PBS with 0.1% BSA (Gibco, PHG0021), and 100 U/mL penicillin, and 100 μg/mL streptomycin (Gibco, 15140‐122). Experiments were performed at pH 7.2 in antibiotic‐containing medium with ≤ 0.6% DMSO. All cells were tested bi‐weekly for mycoplasma contamination and were cultured in a humidified atmosphere in an incubator maintained at 37°C and 5% CO_2_.

### 4.2 Cell Growth and Survival Determination

Two 24‐well plates were seeded overnight in a total volume of 1 mL media per well and incubated overnight at 37°C and 5% CO_2_. The next day, 1 mL of media containing 2× OGM, ERA, or Ogerin was added to wells, and the cells were incubated for 72 h at 37°C and 5% CO_2_. Cells were transferred to 15 mL conicals and the wells were treated with 1 mL of TrypLE Express for 5 min to release any adherent cells (Invitrogen, 12604‐039). Samples were centrifuged at ≥ 300*g* for 5 min, the supernatant removed, and the cell pellet lysed with 200 μL of 1× Passive Lysis Buffer (Promega, PAE1941). Cells were then assessed with CellTiter‐Glo (Promega, PAG7571) using LUMITRAC white‐walled 96‐well microplates (USA Scientific, 5665–5075) on a GloMax‐Multi+ (VWR). Four‐parameter logistic regression curves and LD_50_ calculations were determined using R‐studio.

### 4.3 GSH Assay

Cells were grown and cell pellets collected as described above. Samples were then assessed using the GSH‐Glo Glutathione Assay (Promega, V6911) following the manufacturer's instructions and a black‐walled 96‐well tissue culture plate (VWR; 29444‐008). Plates were read on a GloMax‐Multi+.

### 4.4 FerroOrange Assay

Cells were seeded on six‐well plates and incubated overnight at 37°C and 5% CO_2_. DMSO or OGM was then added to the cells and plates were incubated for 24 h. Cells were then stained with FerroOrange (Dojindo Molecular Technologies Inc., F374) following the manufacturer's instructions for 60 min at 37°C and 5% CO_2_. Cells were washed twice with DPBS (Gibco, 14190144) and imaged in FluoroBrite medium (Gibco, A1896702) on a Cytation C10 (Agilent BioTek) using RFP (Ex 531/40, Em 593/40; 1225101). Quantification was performed blinded (threshold 5000, object size 5–100 μm).

### 4.5 Rescue Experiments

Cells were seeded in 24‐well plates in a total volume of 1 mL medium per well and incubated overnight at 37°C and 5% CO_2_. The following day, 1 mL of medium containing 2× OGM, Fer‐1, Lip‐1, or Z‐VAD‐FMK was added to each well, and cells were incubated for an additional 72 h at 37°C and 5% CO_2_. Cells were transferred to 15 mL conical tubes, and the wells were treated with 1 mL TrypLE Express for 5 min to release any remaining adherent cells. Samples were centrifuged at ≥ 300*g* for 5 min; the supernatant was removed, and the cell pellet was lysed in 200 μL of 1× Passive Lysis Buffer. Lysates were then assessed with CellTiter‐Glo using LUMITRAC white‐walled 96‐well microplates on a GloMax‐Multi+.

### 4.6 Quantitative Reverse Transcription PCR (qRT‐PCR)

Cells were seeded on 100 mm cell culture dishes in 15 mL of media and incubated overnight at 37°C and 5% CO_2_. 2× OGM or ERA in 15 mL of media was added, and the cells were incubated for another 48 h. Cell pellets were collected as described above in a 50 mL conical. Total RNA was collected using the RNeasy Mini Kit (Qiagen, 74106). cDNA was generated using a High‐Capacity cDNA Reverse Transcription Kit with RNase Inhibitor (Applied Biosystems, 4374966), and qRT‐PCR was run using TaqMan Universal Master Mix II, with UNG (Applied Biosystems, 4440045) on a Quant Studio 7 Flex Real‐Time PCR System (Applied Biosystems). Statistical analysis was performed on the Δ*C*_T_ values, and the data were transformed using the $2^{-\Delta \Delta C_{T}}$ method for visualization. TaqMan primers were ordered from ThermoFisher Scientific. The human primers used were GPR68 (Hs00268858_s1), ATF4 (Hs00909569_g1), HMOX1 (Hs01110250_m1), SLC7A11 (Hs00921938_m1), TFRC (Hs00951083_m1), ACTB (Hs01060665_g1), and GAPDH (Hs02786624_g1).

### 4.7 BODIPY 581/591 C11 Staining

Cells were seeded on six‐well plates in media supplemented with 10% FBS (Gibco, A5670701) and incubated overnight at 37°C and 5% CO_2_. DMSO, 2 μM OGM, or 15 μM ERA was then added to the wells, and the plates were incubated for an additional 48 h. Cells were then stained with BODIPY 581/591 C11 (Invitrogen, D3861) for 30 min at 37°C and 5% CO_2_. Cells were washed twice with DPBS (Gibco, 14190144) and imaged in FluoroBrite medium on a Cytation C10 using RFP for non‐oxidized lipids and GFP (Ex 469/35, Em 525/39; 1225101) for oxidized lipids. Quantification was performed blinded (threshold 5000, object size 5–100 μm).

### 4.8 EGFP Transfections

Cells were reverse transfected in a six‐well plate with 5 μg EGFP+ plasmid (VB900139‐8384agr) using Lipofectamine 3000 (Invitrogen, L3000015) in media supplemented with 10% FBS. Cells were gently washed with DPBS, and the media was replaced with FluoroBrite DMEM at 24 h post‐transfection. Cells were then imaged and quantified on a Cytation C10.

### 4.9 GPR68 and ATF4 Overexpressions and Knockdowns

Cells were reverse transfected with Lipofectamine 3000 and 2.5 μg plasmid per well on 12‐well plates in media supplemented with 10% FBS. Samples were collected with 200 μL of 1× Passive Lysis Buffer 72 h post‐transfection and assessed with CellTiter‐Glo using LUMITRAC white‐walled 96‐well microplates. Plates were read on a GloMax‐Multi+. Plasmids were obtained from VectorBuilder. GPR68 shRNAs used in this paper were hGPR68‐shRNA1: 5′‐CCACCGTTGTCACAGACAATG‐3′ (VB221221‐1234czj), hGPR68‐shRNA2: 5′‐GAGCTGTACCATCGACCATAC‐3′ (VB221221‐1235jft) and nontargeting control shRNA (NT‐shRNA): 5′‐CCTAAGGTTAAGTCGCCCTCG‐3′ (VB010000‐9259tcf). ATF4 shRNAs used in this paper were hATF4‐shRNA1: 5′‐CATGATCCCTCAGTGCATAAA‐3′ (VB230508‐1473uhv), hATF4‐shRNA2: 5′‐CCTAGGTCTCTTAGATGATTA‐3′ (VB230508‐1474vhd), and non‐targeting control shRNA: 5′‐CCTAAGGTTAAGTCGCCCTCG‐3′ (VB010000‐9271yez). hGPR68 (VB221110‐1429uwp) with the control plasmid (VB230630‐1409kyc) and hATF4 (VB230104‐1203pag) with the control plasmid (VB900139‐8319ega) were used for overexpression studies.

### 4.10 ATF4 Reporter Assay

Cells were transfected on 12‐well plates as described above with the ATF4 luciferase reporter plasmid from Addgene (plasmid #21850) [42]. Twenty‐four hours post‐transfection, DMSO or 10 μM OGM was added to each well. At 72 h post‐transfection, samples were collected in Passive Lysis Buffer. Cells were then assessed with CellTiter‐Glo and Luciferase Assay Reagent II (Promega, E1910) using LUMITRAC white‐walled 96‐well microplates. Plates were read on a GloMax‐Multi+.

### 4.11 Statistical Analysis and Graphs

Results are given as mean ± standard deviation for all figures except Figures 1F and 3A, which is given as mean ± standard error of the mean. All experiments were conducted with *n* ≥ 3 independent biological replicates to ensure robust and reproducible outcomes. All results are representative of at least technical triplicates. Significant differences between the data of different groups were evaluated by Student's *t*‐test with post hoc Bonferroni correction analysis when appropriate. Significance was denoted by *α* levels of *< 0.05, **< 0.01, and ***< 0.001. Significance was calculated with R 4.4.0, and graphs were made in R‐studio.

## 5 Additional Information

### 5.1 Limitations

This study is focused on two well‐established ATRT cell lines, which may not fully capture the heterogeneity of ATRTs and warrants further study in larger cohorts. In vivo validation, particularly in orthotopic models and patient‐derived xenografts, is needed to confirm the efficacy of OGM and ERA. Furthermore, compound concentrations were empirically determined for each assay, and future pharmacokinetic, toxicity, and in vivo efficacy studies will be required to determine whether therapeutically relevant exposures can be achieved.

## Author Contributions

**Leif R. Neitzel:** conceptualization, data curation, formal analysis, funding acquisition, investigation, methodology, visualization, writing – original draft. **Charles C. Hong:** supervision, funding acquisition, writing – review and editing.

## Funding

Our work was funded by NIH/NHLBI R01HL167133A1 to CCH and Michigan State University's Department of Medicine Seed Funding to LRN.

## Ethics Statement

The authors have nothing to report.

## Consent

The authors have nothing to report.

## Conflicts of Interest

C.C.H. is an inventor on an issued patent related to this manuscript. The other author declares no conflicts of interest.

## Supporting information


**Supplementary data file**: cnr270719‐sup‐0001‐3_Data.xlsx.

## Data Availability

All data used in the analysis and generation of figures are included in this published article as a supplementary data file.
